# Serum anti-Müllerian hormone response to pyrroloquinoline quinone supplementation in healthy women: no overall change and exploratory subgroup findings

**DOI:** 10.3389/fendo.2026.1831604

**Published:** 2026-07-10

**Authors:** Saori Tsuji, Tsuyoshi Takiuchi, Mika Handa, Naoki Miura, Hisae Aoyagi, Yuki Uematsu, Miwako Shidomi, Kazutake Fukada, Chiaki Ogura, Tadashi Kimura, Michiko Kodama

**Affiliations:** 1 Department of Obstetrics and Gynecology, Graduate School of Medicine, The University of Osaka, Suita, Japan; 2 Department of Clinical Genomics, Graduate School of Medicine, The University of Osaka, Suita, Japan; 3 Miura Clinic, Medical Corporation Kaonkai, Osaka, Japan; 4 Strategic Design Headquarters, ROHTO Pharmaceutical Co., Ltd., Tokyo, Japan; 5 Health Skin Science Research Planning Division, ROHTO Pharmaceutical Co., Ltd., Tokyo, Japan; 6 Basic Research and Development Division, ROHTO Pharmaceutical Co., Ltd., Kizugawa, Japan; 7 Internal Medicine and Functional Food Development Division, ROHTO Pharmaceutical Co., Ltd., Osaka, Japan; 8 Sakai City Medical Center, Sakai City Hospital Organization, Sakai, Japan; 9 Department of Children’s and Women’s Health, Division of Health Sciences, The University of Osaka Graduate School of Medicine, Suita, Japan

**Keywords:** anti-Müllerian hormone (AMH), follicular development, ovarian reserve, oxidative stress, pyrroloquinoline quinone (PQQ), reproductive health

## Abstract

**Introduction:**

Oocyte maturation requires substantial ATP, and the resulting oxidative stress may impair follicular development. Pyrroloquinoline quinone (PQQ), an antioxidant compound, protects mitochondria and promotes follicular development in animal models; however, its effects on human ovarian function remain unclear.

**Methods:**

This single-arm, open-label study prospectively evaluated the effects of oral PQQ supplementation on ovarian reserve and related clinical and biochemical outcomes. The primary outcome was serum anti-Müllerian hormone (AMH). Secondary outcomes included luteinizing hormone, follicle-stimulating hormone, estradiol, biological antioxidant potential, reactive oxygen metabolites-derived compounds (d-ROMs), and Menstrual Distress Questionnaire (MDQ) scores. Fifty healthy women aged 25–42 years with regular menstrual cycles and baseline serum AMH levels of 0.5 ≤ AMH < 3.0 ng/mL received 20 mg/day of PQQ for 90 ± 10 days. Blood samples were collected on menstrual cycle days 1–7 before and after supplementation. Exploratory subgroup analyses were performed using combined stratification by age and baseline AMH.

**Results:**

Analyses were conducted in the per-protocol set (*n* = 35). AMH did not change significantly overall (1.561 ± 0.689 vs. 1.439 ± 0.772 ng/mL, *p* = 0.182). In younger participants with lower baseline AMH (*n* = 7), AMH showed a non-significant increase (1.121 ± 0.379 vs. 1.361 ± 0.604 ng/mL, *p* = 0.056), accompanied by a significant decrease in d-ROMs (351.3 ± 43.5 vs. 305.3 ± 34.3 U. CARR, *p* = 0.027). In contrast, older participants with lower baseline AMH (*n* = 13) showed a significant decrease in AMH (1.066 ± 0.296 vs. 0.852 ± 0.312 ng/mL, *p* = 0.033), while d-ROMs remained unchanged. Across the overall cohort, d-ROMs showed a non-significant reduction (334.2 ± 59.7 vs. 321.5 ± 63.9 U. CARR, *p* = 0.090).

**Conclusion:**

PQQ supplementation did not alter AMH levels in the overall cohort. Subgroup findings were inconsistent and should be interpreted cautiously given the small sample sizes and absence of a control group. These exploratory results do not permit conclusions regarding clinical efficacy. Larger, placebo-controlled trials with imaging-based and clinical reproductive endpoints are needed to determine whether PQQ has measurable effects on ovarian biology.

**Clinical trial registration:**

## 1 Introduction

Ovarian function is crucial for female fertility and women’s health across the life course. Declining ovarian reserve contributes to infertility, and national assisted reproductive technology (ART) registry data in Japan indicate that pregnancy outcomes remain modest even with *in vitro* fertilization (IVF) (1). Ovarian dysfunction also imposes a substantial socioeconomic burden; for example, menstruation-related symptoms and menopausal disorders among working women have been estimated to cause an annual economic loss of approximately 362.8 billion JPY in Japan (2). Collectively, these considerations underscore the importance of maintaining ovarian function. Ovarian function is closely linked to ovarian reserve, which reflects the size of the remaining oocyte pool. Oocyte maturation is a complex process that is regulated both hormonally and genetically. At birth, primordial follicles remain in a dormant state, arrested in prophase I of meiosis until the onset of puberty (3). After puberty, follicles develop under gonadotropin stimulation, with granulosa cells acquiring follicle-stimulating hormone (FSH) responsiveness and subsequently luteinizing hormone (LH) receptor expression as follicles mature and 17β-estradiol (E2) production increases (4, 5). During follicle recruitment, most follicles undergo atresia and do not reach ovulation, contributing to the progressive decline in ovarian reserve over time. Anti-Müllerian hormone (AMH) is secreted by granulosa cells of the preantral and small antral follicles and is widely used as a biomarker of ovarian reserve and follicular development (6).

Oocyte maturation requires a large amount of ATP, and the associated increase in mitochondrial activity can enhance oxidative stress and impair follicular development. Because excessive oxidative stress can disrupt folliculogenesis and compromise oocyte quality, antioxidant supplementation has been explored as a potential strategy to support ovarian function; however, clinical evidence remains mixed and appears to depend heavily on the study population and underlying mechanisms. For example, in infertile women with occult premature ovarian insufficiency (OPOI), who have normal menstruation and FSH levels but decreased fecundity, combined supplementation with selenium and vitamin E (selenium as a cofactor for antioxidant selenoenzymes such as glutathione peroxidase, and vitamin E as a lipid-soluble chain-breaking antioxidant that limits lipid peroxidation) led to an increase in serum AMH and mean ovarian volume, suggesting improved follicular activity (7). In contrast, in patients undergoing hysterectomy with bilateral salpingectomy, an intervention reported to reduce AMH levels postoperatively, preoperative supplementation with coenzyme Q10 (CoQ10) (a mitochondrial electron carrier with lipophilic antioxidant activity) did not modify the magnitude of the postoperative decline in AMH levels compared with the placebo (8). These contrasting findings highlight a critical gap: the effects of antioxidant supplementation on ovarian reserve may vary substantially depending on baseline ovarian status, mitochondrial function, and redox balance, yet few studies have systematically examined these factors in healthy women.

Pyrroloquinoline quinone (PQQ) is an antioxidant compound identified as the third redox cofactor alongside nicotinamide (vitamin B3) and riboflavin (vitamin B2) (9). A study in PQQ-deficient mice has revealed impaired growth and abnormal phenotypes, suggesting a potential role as a growth factor or vitamin (10). Although its classification as a vitamin remains controversial, PQQ has been reported to exert beneficial effects on metabolic regulation, inflammation, cognitive function, and cerebral blood flow in mammals, including humans (11–15). However, evidence regarding the effects of PQQ on ovarian function has been limited to animal studies only. In mice, PQQ has been shown to reduce oxidative stress during follicular development, resulting in increased ovulation, fertilization, blastocyst formation, and litter size (16). Despite these promising findings, no clinical study has evaluated whether PQQ influences ovarian reserve or reproductive hormonal profiles in humans, particularly in healthy women with normal menstrual cycles. This represents a notable gap in the literature, particularly given the growing interest in mitochondrial-targeted nutritional interventions for reproductive health. To address this gap, the present study prospectively investigated the effects of PQQ supplementation in healthy women, with the primary objective of assessing changes in serum AMH levels after PQQ supplementation and secondary objectives of evaluating changes in reproductive hormones, oxidative stress markers, and menstrual-related symptoms.

## 2 Materials and methods

### 2.1 Study design and participants

**Figure 1:**
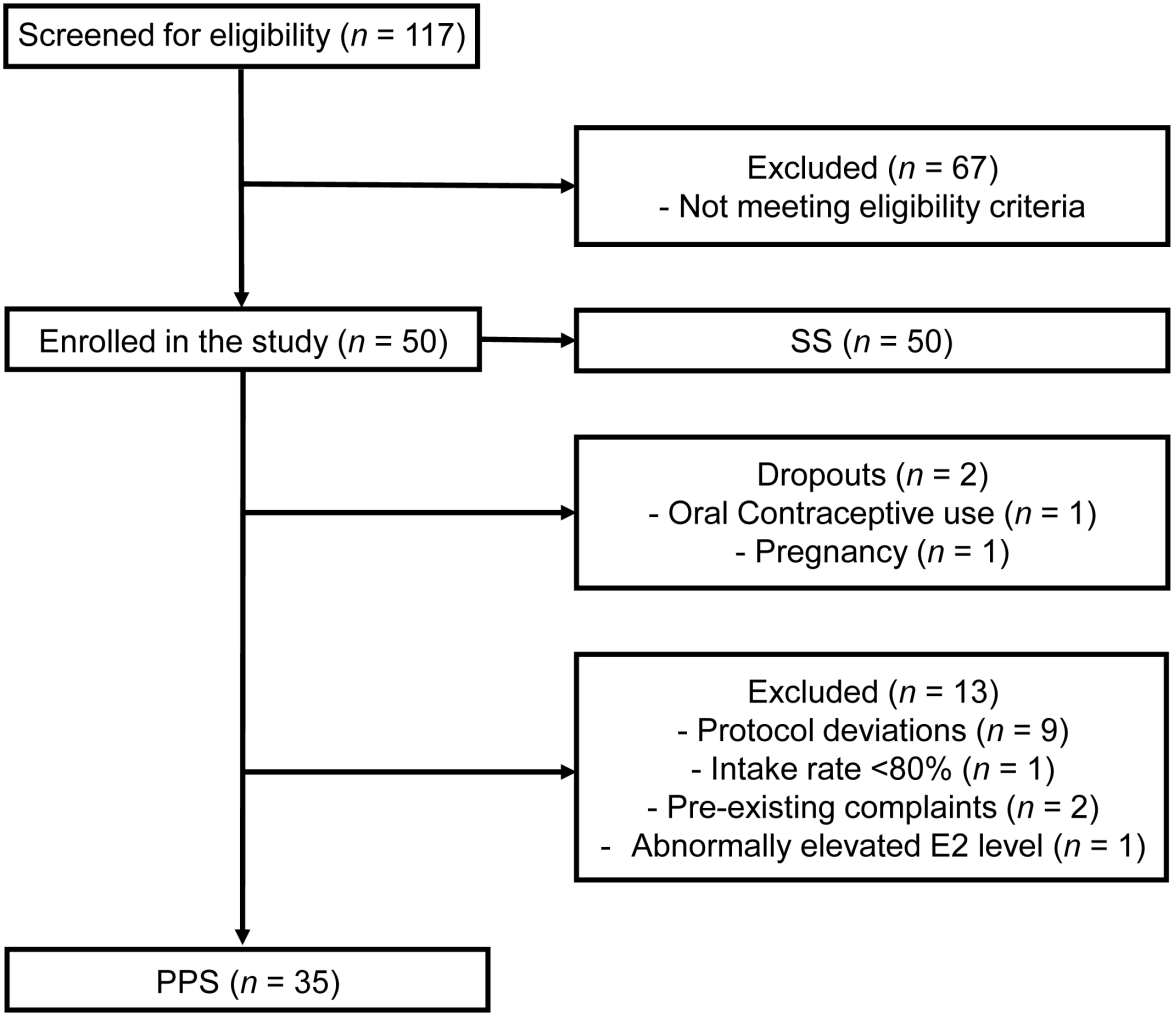
Flowchart of participant selection and definitions of the analysis sets. SS, safety set; E2, estradiol; PPS, per-protocol set.

This single-arm, open-label prospective study was conducted at Miura Clinic, Medical Corporation Kanonkai in Japan from November 24, 2022, to April 30, 2023. This study was conducted in accordance with the Declaration of Helsinki and the Ethical Guidelines for Medical and Biological Research Involving Human Subjects. The protocol was reviewed and approved by the Institutional Review Board of Miura Clinic, Medical Corporation Kanonkai (IRB No. 17000161). This study was prospectively registered in the UMIN Clinical Trials Registry (ID: UMIN000049793; https://center6.umin.ac.jp/cgi-open-bin/ctr_e/ctr_view.cgi?recptno=R000056669). A flow diagram of participant enrollment and analysis sets is provided in **Figure 1**. The participants were healthy women aged 25–42 years with regular menstrual cycles and baseline serum AMH levels of 0.5 ≤ AMH < 3.0 ng/mL. Individuals were excluded if they had a history of cardiac, hepatic, renal, or metabolic disease; known allergies; anemia; were pregnant or breastfeeding; intended to conceive during the study period; or were currently using female sex hormone medications, PQQ, or CoQ10 supplements. Additional exclusion criteria included intense exercise routines, adherence to restrictive or irregular dietary patterns, alcohol consumption >60 g/day, smoking, a body mass index (BMI) ≥30 kg/m^2^, and concurrent participation in other clinical trials. Participants could withdraw at any time. The investigator could discontinue participation for safety reasons (including a serious adverse event), pregnancy, an intercurrent illness that could affect study outcomes, or other reasons deemed necessary. All participants provided written informed consent before enrollment. This manuscript was prepared with reference to CONSORT principles and the TREND statement, as applicable to a single-arm, non-randomized exploratory intervention study, in accordance with EQUATOR Network recommendations.

### 2.2 Procedures

Screening examinations were conducted on menstrual cycle days 1–7 (early follicular phase) to assess eligibility for study enrollment. The screening process included a comprehensive medical questionnaire, the Menstrual Distress Questionnaire (MDQ), and the Profile of Mood States 2nd Edition (POMS2). The MDQ is used to assess menstrual-related symptoms across 8 subscales (pain, impaired concentration, behavioral changes, autonomic dysregulation, fluid retention, negative affect, mood elevation, and perceived control), with each item rated on a four-point scale ranging from 0 (no symptoms) to 3 (severe symptoms) (17). The POMS2 is used to assess transient affective states across 7 subscales (Anger-Hostility, Confusion-Bewilderment, Depression-Dejection, Fatigue-Inertia, Tension-Anxiety, Vigor-Activity, and Friendliness). A Total Mood Disturbance (TMD) score is calculated by subtracting the Vigor score from the sum of the negative mood subscale scores, with higher values indicating greater mood disturbance (18). In addition, participants underwent physical examination (blood pressure and pulse measurements) and blood and urine tests. Individuals who met all the inclusion criteria and none of the exclusion criteria were formally enrolled. Data collected during screening were used as baseline values for subsequent comparisons. Each participant was instructed to take 20 mg of pyrroloquinoline quinone disodium salt (PQQ) once daily for 90 ± 10 days and record their PQQ intake in a web-based lifestyle diary. The investigational product (RP-001) provided by ROHTO Pharmaceutical Co., Ltd. was a soft-gel capsule containing 20 mg of PQQ per capsule. The capsule consisted of safflower oil as the vehicle, with gelatin, glycerin, glycerin fatty acid esters, and caramel coloring as excipients (lot number: 1C1A). The capsules were stored in a sealed container in a cool place, avoiding direct sunlight, high temperatures, and humidity. Participants were instructed to take one capsule once daily after breakfast. If a dose was missed, participants were instructed not to take the missed dose on the following day. During the study period, the participants were instructed not to make major changes to their pre-trial lifestyle habits. The use of pharmaceutical drugs was, in principle, prohibited. Participants who had been using supplements other than PQQ or CoQ10 prior to the start of the study were allowed to continue their use during the study period. Initiation of new supplements during the study period was discouraged. Post-supplementation assessments were scheduled for the day following the final dose, timed to coincide with menstrual cycle days 1–7, mirroring the baseline assessment window. Blood samples were collected on menstrual cycle days 1–7 at both the baseline and post-supplementation visits. Adherence was assessed using the diary and by verifying the number of capsules consumed based on the remaining capsules collected at the end of the study; the PQQ intake rate (%) was calculated as (actual number of capsules taken/scheduled number of capsules from the start of supplementation to the day before the final testing) × 100. Participants were also instructed to report any adverse events and concomitant medication use throughout the study period.

### 2.3 Outcomes

The primary outcome was serum AMH level measured at baseline and post-supplementation. Secondary outcomes included serum LH, FSH, and E2 levels; plasma biological antioxidant potential (BAP) and reactive oxygen metabolites-derived compounds (d-ROMs); and MDQ scores. Serum AMH levels were measured using an electrochemiluminescence immunoassay (ECLIA) on the cobas 8000 e801 analyzer (Roche Diagnostics K.K., Tokyo, Japan) with the Elecsys^®^ AMH Plus assay. The analytical range was 0.01–23 ng/mL, and the limit of detection was 0.01 ng/mL. Within-run precision was ≤10%. Serum LH, FSH, and E2 levels were measured using a chemiluminescent immunoassay (CLIA) on the Architect i2000 SR analyzer (Abbott Japan LLC, Tokyo, Japan). The assays used were the Architect^®^ LH II, Architect^®^ FSH, and Architect^®^ Estradiol II kits, respectively. The analytical ranges were 0.09–250.00 mIU/mL (up to 1000.00 mIU/mL with automatic dilution) for LH, 0.05–150.00 mIU/mL (up to 750.00 mIU/mL with automatic dilution) for FSH, and 10–1000 pg/mL (up to 5000 pg/mL with automatic dilution) for E2. All hormone measurements were performed by LSI Medience Corporation, Tokyo, Japan. Plasma BAP and d-ROMs levels were measured in heparinized plasma using a colorimetric method with the FREE Carrio Duo analyzer (Wismer Co., Ltd., Tokyo, Japan). The assays used were the BAP TEST [FOR FREE] (R1, R2) and d-ROMs [FOR FREE] (R1, R2) reagents (Wismer Co., Ltd., Tokyo, Japan). The analytical ranges were 500–6000 µM for BAP and 40–1000 U. CARR for d-ROMs. All analyses were performed by Japan Institute for the Control of Aging, NIKKEN SEIL Co., Ltd., Tokyo, Japan. The laboratory personnel were blinded to all study-related information, including the study objectives, participant characteristics, and the timing of sample collection. Exploratory outcomes included POMS2 scores. MDQ and POMS2 were self-administered questionnaires collected at baseline and post-supplementation. MDQ scores were calculated according to the instrument manual and were analyzed for the premenstrual, menstrual, and postmenstrual phases (total scores). POMS2 subscale scores and the TMD score were calculated according to the manual. MDQ and POMS2 were compared between baseline and post-supplementation. Safety assessments comprised blood tests, urine tests, and physical examinations. Adverse events were collected throughout the study period (from the first dose until the final visit) and were assessed by the investigator for severity and relationship to PQQ intake according to predefined criteria.

### 2.4 Sample size

As this was an exploratory study and prior data on expected AMH responses to PQQ were unavailable, no formal sample size calculation was performed. We planned to enroll 50 participants to obtain approximately 40 evaluable participants, based on feasibility considerations and the sample sizes commonly used in similar exploratory studies in this field (19, 20).

### 2.5 Statistical analysis

#### 2.5.1 Analysis sets

The participants were categorized into the following analysis sets. Given the exploratory, single-arm design, the per-protocol set (PPS) was prespecified as the primary analysis population to evaluate outcomes under adequate adherence to the intervention.

Per-protocol set (PPS):

Participants who received PQQ, had at least one efficacy-related data point, and adhered sufficiently to the study protocol. Participants were excluded from the PPS if they met any of the following criteria:

1. PQQ intake rate <80% (as defined in Section 2.2).
2. Post-enrollment ineligibility.
3. Noncompliance with study restrictions.
4. Major protocol deviations.
5. Determined by the principal investigator to be inappropriate for inclusion in the PPS.

This set was used for analyses of hormonal and psychological outcomes.

Safety set (SS):

All participants who received at least one dose of PQQ. This set was used for analyses of safety and baseline characteristics.

#### 2.5.2 Statistical methods

Statistical analyses were conducted using IBM SPSS version 28 (IBM Japan, Ltd.) and JMP 12 (SAS Institute Japan, Ltd.). Continuous variables were summarized as mean ± SD (or median [interquartile range (IQR)], as appropriate). For hormonal and oxidative stress markers, the normality of paired differences (post–pre) was assessed using Shapiro-Wilk test. When normality was confirmed, paired *t*-tests were applied; when normality was not met, Wilcoxon signed-rank tests were used. MDQ and POMS2 scores were analyzed using Wilcoxon signed-rank tests. All tests were two-sided, and statistical significance was defined as *p* < 0.05. Results with 0.05 < *p* < 0.10 were interpreted as non-significant exploratory findings, and their numerical direction was described using neutral terminology (e.g., “non-significant increase/decrease”). No imputation was performed; analyses were conducted using available data. Subgroup analyses were performed to explore the differential effects of PQQ supplementation on the primary outcome (serum AMH levels). Participants were stratified into two subgroups (below vs. at or above the median) based on each of the following baseline parameters: age, AMH, BAP, and d-ROMs. In addition, a combined stratification based on age and AMH was used to create four subgroups: younger age with lower AMH, younger age with higher AMH, older age with lower AMH, and older age with higher AMH. To further explore potential mechanisms of action, subgroup analyses of d-ROMs as a secondary efficacy endpoint were conducted using the same four combined age-AMH categories. Subgroup analyses of LH, FSH, and E2 were conducted, excluding participants whose menstrual cycle day at testing differed by more than five days between pre- and post-supplementation assessments, since these hormone levels vary according to the menstrual cycle day on which blood samples are collected. Changes (post–pre) were calculated; within each subgroup, the mean change was tested against zero using one-sample *t*-tests (with zero change as the reference). All primary and secondary analyses were prespecified. Subgroup analyses were exploratory, and no adjustment for multiplicity was performed.

## 3 Results

### 3.1 Participant characteristics

A total of 117 participants provided written informed consent and were screened for eligibility. Of these, 67 were excluded for not meeting the eligibility criteria, and 50 participants who met all inclusion criteria and none of the exclusion criteria were enrolled. The flow of participant selection and the derivation of the PPS and SS are illustrated in **Figure 1**. After study initiation, two participants voluntarily withdrew and were considered dropouts. Among the remaining 48 participants, 13 were excluded from the PPS according to predefined criteria (**Figure 1**), resulting in 35 participants in the PPS. The SS included all 50 participants who were enrolled in the study. Baseline characteristics of the participants are summarized in **Table 1**. The median (IQR) age was 38.5 (33.75–41) years and the mean PQQ intake rate in the PPS was 98.12 ± 0.03%.

**Table 1:** Participant characteristics in the SS (*n* = 50).

Characteristic	Screening and pre-supplementation
Age (years)	37.1 ± 4.5
Height (cm)	158.89 ± 4.65
Weight (kg)	54.04 ± 7.37
BMI (kg/m^2^)	21.40 ± 2.72
Systolic blood pressure (mmHg)	116.0 ± 12.5
Diastolic blood pressure (mmHg)	75.2 ± 11.2
Pulse (beats/minute)	73.92 ± 11.4

Values are presented as mean ± SD.

SS, safety set; BMI, body mass index.

### 3.2 Changes in hormone levels and oxidative stress markers

Changes in hormone levels before and after PQQ supplementation are presented in **Table 2**. The median baseline AMH level (1.555 ng/mL) was used as the cutoff for subgroup analyses. AMH levels did not significantly change after supplementation (1.561 ± 0.689 vs. 1.439 ± 0.772 ng/mL, *p* = 0.182). FSH levels showed a non-significant increase (6.370 ± 2.330 vs. 7.287 ± 3.757 mIU/mL, *p* = 0.088), whereas LH and E2 levels did not significantly change. In a sensitivity analysis excluding two participants whose menstrual cycle day at testing differed by more than five days between the pre- and post-supplementation assessments, FSH levels significantly increased (*n* = 33, 6.286 ± 2.367 vs. 7.423 ± 3.807 mIU/mL, *p* = 0.026) (**Supplementary Table 1**). Changes in oxidative stress markers are shown in **Table 3**. D-ROMs levels showed a non-significant decrease (334.2 ± 59.7 vs. 321.5 ± 63.9 U. CARR, *p* = 0.090), whereas BAP levels did not significantly change (2164.7 ± 136.4 vs. 2145.4 ± 173.6 µM, *p* = 0.548).

**Table 2:** Changes in hormone levels before and after PQQ supplementation.

Hormone	Pre-supplementation	Post-supplementation	*p*-value
AMH (ng/mL)	1.561 ± 0.689	1.439 ± 0.772	0.182
LH (mIU/mL)	2.999 ± 1.212	3.413 ± 2.395	0.844
FSH (mIU/mL)	6.370 ± 2.330	7.287 ± 3.757	0.088^†^
E2 (pg/mL)	64.8 ± 33.4	78.9 ± 110.5	0.166

Values are presented as mean ± SD. Statistical comparisons were conducted using paired *t*-tests or Wilcoxon signed-rank tests, depending on the normality of paired differences (post–pre). ^†^0.05 < *p* < 0.10.

PQQ, pyrroloquinoline quinone; AMH, anti-Müllerian hormone; LH, luteinizing hormone; FSH, follicle-stimulating hormone; E2, estradiol.

**Table 3:** Changes in oxidative stress markers before and after PQQ supplementation.

Marker	Pre-supplementation	Post-supplementation	*p*-value
BAP (µM)	2164.7 ± 136.4	2145.4 ± 173.6	0.548
d-ROMs (U. CARR)	334.2 ± 59.7	321.5 ± 63.9	0.090^†^

Values are presented as mean ± SD. Statistical comparisons were conducted using paired *t*-tests or Wilcoxon signed-rank tests, depending on the normality of paired differences (post–pre). ^†^0.05 < *p* < 0.10.

PQQ, pyrroloquinoline quinone; BAP, biological antioxidant potential; d-ROMs, reactive oxygen metabolites-derived compounds.

Subgroup analyses of AMH stratified by age, baseline AMH, BAP, d-ROMs, and the combined age–baseline AMH categories revealed distinct response patterns (**Table 4**). In older participants, AMH levels significantly decreased (*n* = 20, *p* = 0.020). A non-significant decrease was observed among participants with lower baseline BAP (*n* = 19, *p* = 0.089) and lower baseline d-ROMs (*n* = 18, *p* = 0.054). In younger participants with lower baseline AMH levels, AMH showed a non-significant increase (*n* = 7, *p* = 0.056), whereas in older participants with lower baseline AMH levels, AMH significantly decreased (*n* = 13, *p* = 0.033). In this younger low-AMH subgroup, d-ROMs levels also decreased significantly following supplementation (*n* = 7, *p* = 0.027) (**Table 5**).

**Table 4:** Subgroup analyses of serum AMH levels (ng/mL) before and after PQQ supplementation.

Subgroup	*n*	Pre-supplementation	Post-supplementation	*p*-value
Younger age	15	1.648 ± 0.661	1.673 ± 0.760	0.844
Older age	20	1.497 ± 0.720	1.263 ± 0.751	0.020*
Lower AMH	20	1.086 ± 0.318	1.031 ± 0.489	0.514
Higher AMH	15	2.196 ± 0.511	1.983 ± 0.753	0.257
Lower BAP	19	1.540 ± 0.761	1.328 ± 0.800	0.089^†^
Higher BAP	16	1.587 ± 0.616	1.570 ± 0.741	0.904
Lower d-ROMs	18	1.782 ± 0.617	1.522 ± 0.754	0.054^†^
Higher d-ROMs	17	1.328 ± 0.701	1.351 ± 0.804	0.850
Younger age with lower AMH	7	1.121 ± 0.379	1.361 ± 0.604	0.056^†^
Younger age with higher AMH	8	2.109 ± 0.480	1.946 ± 0.812	0.454
Older age with lower AMH	13	1.066 ± 0.296	0.852 ± 0.312	0.033*
Older age with higher AMH	7	2.296 ± 0.564	2.026 ± 0.740	0.437

Participants were stratified into two subgroups (below vs. at or above the median) based on each baseline parameter (age, AMH, BAP, and d-ROMs), and into four subgroups based on age and baseline AMH levels (younger age with lower AMH, younger age with higher AMH, older age with lower AMH, and older age with higher AMH). Values are presented as mean ± SD. Changes (post–pre) were calculated, and within each subgroup the mean change was tested against zero using one-sample *t*-tests or Wilcoxon signed-rank tests, depending on the normality of within-subgroup changes (post–pre). *p < 0.05; ^†^0.05 < *p* < 0.10.

PQQ, pyrroloquinoline quinone; AMH, anti-Müllerian hormone; BAP, biological antioxidant potential; d-ROMs, reactive oxygen metabolites-derived compounds.

**Table 5:** Changes in serum d-ROMs levels (U. CARR) before and after PQQ supplementation stratified by age and baseline AMH levels.

Subgroup	*n*	Pre-supplementation	Post-supplementation	*p*-value
Younger age with lower AMH	7	351.3 ± 43.5	305.3 ± 34.3	0.027*
Younger age with higher AMH	8	338.9 ± 80.1	336.9 ± 84.5	0.830
Older age with lower AMH	13	320.7 ± 61.7	320.6 ± 74.0	0.995
Older age with higher AMH	7	336.7 ± 49.6	321.6 ± 45.1	0.342

Values are presented as mean ± SD. Changes (post–pre) were calculated, and within each subgroup the mean change was tested against zero using one-sample *t*-tests or Wilcoxon signed-rank tests, depending on the normality of within-subgroup changes (post–pre). *p < 0.05.

PQQ, pyrroloquinoline quinone; d-ROMs, reactive oxygen metabolites-derived compounds; AMH, anti-Müllerian hormone.

### 3.3 Changes in MDQ and POMS2 scores

Changes in MDQ and POMS2 scores before and after PQQ supplementation are presented in **Table 6** and **Supplementary Table 2**, respectively. Postmenstrual MDQ scores significantly increased after PQQ supplementation (*p* < 0.001). No significant changes were observed in the MDQ scores during the premenstrual or menstrual phases. Regarding POMS2, Confusion-Bewilderment (CB) and Vigor-Activity (VA) scores showed non-significant increases (*p* = 0.055 and 0.052, respectively), while no significant changes were observed in the other subscales.

**Table 6:** Changes in MDQ scores before and after PQQ supplementation across three menstrual phases: premenstrual, menstrual, and postmenstrual phase.

Menstrual phase	Pre-supplementation	Post-supplementation	*p*-value
Premenstrual phase	27.2 ± 26.7	23.0 ± 21.7	0.591
Menstrual phase	26.8 ± 24.6	30.5 ± 27.4	0.360
Postmenstrual phase	7.1 ± 11.9	18.2 ± 22.6	<0.001***

Values are presented as mean ± SD. Statistical comparisons were conducted using Wilcoxon signed-rank test. ***p < 0.001.

PQQ, pyrroloquinoline quinone; MDQ, Menstrual Distress Questionnaire.

### 3.4 Safety assessment

A total of 99 adverse events were reported, including 65 mild and 34 moderate events. No serious adverse events were observed. A mild adverse event involving elevated E2 levels (2740 pg/mL) could not be followed up due to the participant’s circumstances; however, other potential causes, such as a possible pregnancy at the time of the final testing, were considered more likely, and the relationship of the event with PQQ intake was judged as “unlikely related.” All other events were either mild or moderate in severity and were assessed to be unrelated to PQQ intake. No cases of side effects attributable to the supplement were identified.

## 4 Discussion

In this study, continuous supplementation of PQQ (20 mg/day for approximately 90 days) did not result in significant changes in serum AMH levels in the overall cohort of healthy women. In exploratory subgroup analyses, younger participants with lower baseline AMH showed a non-significant increase in AMH accompanied by reduced d-ROMs, whereas older participants with lower baseline AMH exhibited a significant decrease. A modest increase in FSH was observed after supplementation; however, because FSH is generally a less robust marker of ovarian reserve than AMH (6) and AMH did not change in the overall cohort, its physiological and clinical significance remains uncertain. Regarding psychological and menstrual-related outcomes, menstrual and premenstrual MDQ scores and POMS2 scores did not change, whereas postmenstrual MDQ scores increased significantly. 

Previous clinical studies evaluating the efficacy of antioxidant supplementation other than PQQ in improving ovarian function have yielded inconsistent results. Improvements in ovarian reserve markers have been reported in patients with OPOI receiving selenium and vitamin E (7) and in patients with PCOS receiving ellagic acid or oleoylethanolamide (20, 21), whereas CoQ10 did not prevent the postoperative decline in AMH after hysterectomy with bilateral salpingectomy (8). The lack of efficacy in the CoQ10 study has been attributed to factors such as lower dosage, shorter duration of supplementation, and mechanisms other than oxidative stress (8, 22), suggesting that antioxidant supplementation may not influence ovarian reserve when oxidative stress is not the primary mechanism. These discrepancies highlight how differences in study design, oxidative status and biological context may influence outcomes. Because oxidative stress has been implicated in the pathophysiology of POI and PCOS (23–27), antioxidant-based interventions may be more relevant in these populations than in healthy women, whose oxidative status is heterogenous and in whom ovarian function may be influenced by factors unrelated to oxidative stress. Accordingly, the observed subgroup findings should be interpreted as exploratory rather than evidence of a definitive treatment effect.

Although AMH is generally stable across the menstrual cycle and exhibits limited short-term variability (28), modest intra-individual fluctuations and assay variability can still occur (29). In addition, the small sample size, single-arm design, and absence of adjustment for multiplicity increase the likelihood that apparent subgroup differences—particularly the decrease in older participants—reflect regression to the mean, assay variability, or random variation rather than a true biological effect. Because AMH reflects only the number of small growing follicles and does not capture oocyte quality or overall reproductive potential (30), short-term changes may arise from multiple non-biological or physiological sources. Moreover, AMH has limited value for predicting natural fertility despite its utility in forecasting ovarian response in IVF cycles (31), and this study did not assess antral follicle count (AFC), ovulation, or clinical reproductive outcomes. Therefore, the divergent AMH patterns observed in the younger and older low-AMH subgroups should be regarded strictly as exploratory and cannot be used to infer changes in fertility potential.

CoQ10 has been extensively studied as a mitochondrial nutrient and has been reported to improve oocyte mitochondrial function, ATP production, and ovarian reserve, particularly in the context of reproductive aging (32, 33). In contrast, PQQ promotes mitochondrial biogenesis through PGC-1α-related pathways and modulates oxidative stress, suggesting a mechanistically distinct and potentially complementary role (34). Although the present study did not evaluate ART-related or conception outcomes, the hormonal and oxidative stress patterns observed here provide preliminary justification for further controlled trials to determine whether the exploratory signals observed in this study could translate into measurable reproductive outcomes.

We set a daily PQQ dose of 20 mg, consistent with amounts used in prior human studies (14, 35) and with doses contained in foods with functional claims. Although PQQ has been reported to support mitochondrial biogenesis and redox homeostasis, the extent to which these mechanisms translate into measurable changes in ovarian biomarkers over short timeframes remains unclear. In mice, PQQ supplementation has been reported to increase ovulation, fertilization, blastocyst formation, and litter size in a dose-dependent manner (16), whereas extremely high doses may exhibit cytotoxic effects (36). Although some commercially available supplements in certain countries contain PQQ up to 50 mg, previous clinical trials have demonstrated the safety and efficacy of daily intake up to 20 mg (14, 35). Given that ovarian folliculogenesis spans approximately one year (37), short-term supplementation may be insufficient to influence AMH or other reserve markers, particularly in older individuals. Therefore, dose-ranging and longer-duration studies within established safety boundaries are needed to clarify whether different dosing strategies may be required to affect ovarian biomarkers.

Because d-ROMs reflect oxidative stress generation whereas BAP represents endogenous antioxidant capacity, these markers capture different aspects of redox biology and may not change in parallel. The reduction in d-ROMs observed in younger participants with lower AMH may suggest a short-term decrease in oxidative stress; however, this interpretation remains speculative given the exploratory nature of the subgroup analyses and the absence of a control group. Endogenous antioxidant capacity, as reflected by BAP, might require longer-term or more substantial shifts in redox homeostasis to show measurable change. Accordingly, the divergence between d-ROMs and BAP should be interpreted cautiously.

Regarding patient-reported outcomes, postmenstrual MDQ scores significantly increased after supplementation, while premenstrual and menstrual phase scores were unchanged. Because the postmenstrual subscale of MDQ primarily captures general psychological and somatic symptoms—such as fatigue, concentration difficulties, and irritability—rather than menstrual-specific complaints, this increase may not indicate worsening menstrual symptoms. Non-significant increases in POMS2 VA and CB scores may reflect transient changes in arousal or cognitive clarity, aligning with previous reports linking PQQ supplementation to improvements in attention, cognitive processing, sleep quality, and fatigue (35, 38, 39). However, psychological measures are subjective and susceptible to contextual influences, and the study was not powered to detect changes in these outcomes. Therefore, the clinical relevance of these changes remains uncertain and warrants confirmation with objective measures of sleep, fatigue, and cognition.

This study has several limitations. First, no formal power calculation was performed because the study was designed as an exploratory, single-arm nutritional intervention and prior data on expected AMH response to PQQ were unavailable. As a result, the overall sample size—and particularly the small subgroups (e.g., *n* = 7 in the younger participants with lower AMH and *n* = 13 in the older participants with lower AMH)—substantially limits statistical power and increases the likelihood that apparent changes may reflect regression to the mean, assay variability, or random variation rather than true biological effects. Second, although AMH is generally stable across the menstrual cycle (28), modest intra-individual variation can still occur (29), and short-term changes should be interpreted as associative rather than causal. Blood sampling within cycle days 1–7 may also have introduced hormonal variability. Third, ovarian reserve was assessed primarily by hormones without imaging-based or clinical endpoints, and AMH itself reflects only the pool of small growing follicles, does not capture oocyte quality or overall reproductive potential (30). Finally, the intervention dose and duration may not capture longer-term or dose-dependent effects, particularly given the long duration of folliculogenesis (37), and the study was not powered to detect changes in psychological outcomes.

Despite these limitations, this study represents the first clinical investigation of PQQ supplementation in humans in relation to ovarian function and provides preliminary data to inform the design of adequately powered controlled trials with more comprehensive outcome measures. In addition, the within-subject pre–post design helped minimize inter-individual variability in hormonal and oxidative stress markers. Furthermore, the inclusion of d-ROMs and BAP—markers rarely incorporated into nutritional intervention studies targeting ovarian reserve—provide exploratory mechanistic insight beyond the existing literature.

Future research should employ randomized, placebo-controlled trials with larger sample sizes and prespecified stratification by age, baseline AMH, and oxidative stress status to clarify which specific subgroups respond differentially to PQQ supplementation. These subgroup patterns may help guide prevention-oriented trials focusing on younger women with early declines in AMH accompanied by oxidative stress. Such trials should standardize the timing of blood sampling and incorporate imaging-based and clinical outcomes, including AFC, ovulation rate, oocyte quality, and pregnancy outcomes, because hormonal markers alone cannot fully capture ovarian reserve or reproductive potential. Longer-duration and dose-ranging studies with careful safety monitoring, are also warranted, given the long duration of human folliculogenesis and the limited clinical evidence for PQQ in reproductive medicine.

In conclusion, PQQ supplementation did not change serum AMH levels in the overall cohort of healthy women. Exploratory analyses indicated age- and reserve-related differences, including a non-significant numerical increase in AMH accompanied by reduced oxidative stress in younger participants with lower AMH. These preliminary findings should be interpreted with caution. Adequately powered, placebo-controlled clinical trials are required to determine whether these exploratory findings represent true biological effects and to clarify any potential clinical relevance.

## Data Availability

The datasets presented in this article are not readily available because the datasets generated and analyzed in this study are held by ROHTO Pharmaceutical Co., Ltd. Individual-level raw data cannot be shared due to confidentiality policies and the absence of participant consent for external disclosure. Only aggregated or clarified information about the analyses may be provided upon reasonable request, subject to approval by ROHTO Pharmaceutical Co., Ltd. Requests to access the datasets should be directed to TT, takiuchi.tsuyoshi.med@osaka-u.ac.jp.
