# Supplementary material for: Serum anti-Müllerian hormone response to pyrroloquinoline quinone supplementation in healthy women: no overall change and exploratory subgroup findings
Source: Front Endocrinol (Lausanne). 2026 Jul 10;17:1831604. doi: 10.3389/fendo.2026.1831604 (PMC13395729; doi:10.3389/fendo.2026.1831604)
Supplement: Supplementary file 1 [file Table1.docx]

**Supplementary Table S1.**

Changes in LH, FSH, and E2 levels before and after PQQ supplementation in participants whose menstrual cycle day at testing differed by ≤5 days between the baseline and post-supplementation visits.

|  | Pre-supplementation | Post-supplementation | *p*-value |
| --- | --- | --- | --- |
| LH (mIU/mL) | 2.870 ± 1.067 | 3.551 ± 2.396 | 0.480 |
| FSH (mIU/mL) | 6.286 ± 2.367 | 7.423 ± 3.807 | 0.026* |
| E2 (pg/mL) | 62.8 ± 30.5 | 79.3 ± 113.4 | 0.260 |

Values are presented as mean ± SD. Statistical comparisons were conducted using paired *t*-tests or Wilcoxon signed-rank tests, depending on the normality of pre-supplementation values.

**p* < 0.05

Abbreviations: PQQ, pyrroloquinoline quinone; LH, luteinizing hormone; FSH, follicle-stimulating hormone; E2, estradiol.
