# Supplementary material for: Serum anti-Müllerian hormone response to pyrroloquinoline quinone supplementation in healthy women: no overall change and exploratory subgroup findings
Source: Front Endocrinol (Lausanne). 2026 Jul 10;17:1831604. doi: 10.3389/fendo.2026.1831604 (PMC13395729; doi:10.3389/fendo.2026.1831604)
Supplement: Supplementary file 2 [file Table2.docx]

**Supplementary Table S2**.

Changes in POMS2 scores before and after PQQ supplementation across TMD and 7 mood subscales: AH, CB, DD, FI, TA, VA, and F.

|  | Pre-supplementation | Post-supplementation | *p*-value |
| --- | --- | --- | --- |
| TMD | 47.2 ± 12.9 | 47.5 ± 11.7 | 0.743 |
| AH | 48.0 ± 13.7 | 48.9 ± 12.9 | 0.582 |
| CB | 42.0 ± 12.4 | 44.9 ± 10.0 | 0.055^†^ |
| DD | 48.7 ± 12.2 | 48.4 ± 11.3 | 0.783 |
| FI | 45.8 ± 10.9 | 47.5 ± 10.3 | 0.313 |
| TA | 45.3 ± 12.6 | 46.2 ± 11.4 | 0.578 |
| VA | 44.2 ± 9.4 | 47.9 ± 10.5 | 0.052^†^ |
| F | 45.0 ± 11.4 | 45.7 ± 8.5 | 0.569 |

Values are presented as mean ± SD. Statistical comparisons were conducted using Wilcoxon signed-rank tests. ^†^0.05 < *p* < 0.10

Abbreviations: PQQ, pyrroloquinoline quinone; POMS2, Profile of Mood States 2nd Edition; TMD, Total Mood Disturbance; AH, Anger-Hostility; CB, Confusion-Bewilderment; DD, Depression-Dejection; FI, Fatigue-Inertia; TA, Tension-Anxiety; VA, Vigor-Activity; F, Friendliness.
